# iCLIP identifies novel roles for SAFB1 in regulating RNA processing and neuronal function

**DOI:** 10.1186/s12915-015-0220-7

**Published:** 2015-12-22

**Authors:** Caroline Rivers, Jalilah Idris, Helen Scott, Mark Rogers, Youn-Bok Lee, Jessica Gaunt, Leonidas Phylactou, Tomaz Curk, Colin Campbell, Jernej Ule, Michael Norman, James B. Uney

**Affiliations:** Regenerative Medicine Laboratories, School of Clinical Sciences, Cellular & Molecular Medicine, Medical Sciences Building, University Walk, University of Bristol, Bristol, BS8 1TD UK; Institute of Medical Sciences & Technology, University of Kuala Lumpur, Kuala Lumpur, 43000 Malaysia; Intelligent Systems Laboratory, Department of Engineering & Mathematics, Merchant Venturers Building, University of Bristol, Bristol, BS8 1UB UK; MRC Centre for Neurodegeneration Research, King’s College London, Institute of Psychiatry, London, UK; Faculty of Computer and Information Science, University of Ljubljana, Trzaska cesta 25, SI-1001 Ljubljana, Slovenia; The Cyprus Institute of Neurology & Genetics, PO Box 23462, 1683 Nicosia, Cyprus; Department of Molecular Neuroscience, UCL Institute of Neurology, Queen Square, London, WC1N 3BG UK

**Keywords:** hnRNP, iCLIP, Long non-coding RNA, miRNA, NCAM1, Neuronal, RNA, SAFB1, Splicing

## Abstract

**Background:**

SAFB1 is a RNA binding protein implicated in the regulation of multiple cellular processes such as the regulation of transcription, stress response, DNA repair and RNA processing. To gain further insight into SAFB1 function we used iCLIP and mapped its interaction with RNA on a genome wide level.

**Results:**

iCLIP analysis found SAFB1 binding was enriched, specifically in exons, ncRNAs, 3’ and 5’ untranslated regions. SAFB1 was found to recognise a purine-rich GAAGA motif with the highest frequency and it is therefore likely to bind core AGA, GAA, or AAG motifs. Confirmatory RT-PCR experiments showed that the expression of coding and non-coding genes with SAFB1 cross-link sites was altered by SAFB1 knockdown. For example, we found that the isoform-specific expression of neural cell adhesion molecule (NCAM1) and ASTN2 was influenced by SAFB1 and that the processing of miR-19a from the miR-17-92 cluster was regulated by SAFB1. These data suggest SAFB1 may influence alternative splicing and, using an NCAM1 minigene, we showed that SAFB1 knockdown altered the expression of two of the three NCAM1 alternative spliced isoforms. However, when the AGA, GAA, and AAG motifs were mutated, SAFB1 knockdown no longer mediated a decrease in the NCAM1 9–10 alternative spliced form. To further investigate the association of SAFB1 with splicing we used exon array analysis and found SAFB1 knockdown mediated the statistically significant up- and downregulation of alternative exons. Further analysis using RNAmotifs to investigate the frequency of association between the motif pairs (AGA followed by AGA, GAA or AAG) and alternative spliced exons found there was a highly significant correlation with downregulated exons. Together, our data suggest SAFB1 will play an important physiological role in the central nervous system regulating synaptic function. We found that SAFB1 regulates dendritic spine density in hippocampal neurons and hence provide empirical evidence supporting this conclusion.

**Conclusions:**

iCLIP showed that SAFB1 has previously uncharacterised specific RNA binding properties that help coordinate the isoform-specific expression of coding and non-coding genes. These genes regulate splicing, axonal and synaptic function, and are associated with neuropsychiatric disease, suggesting that SAFB1 is an important regulator of key neuronal processes.

**Electronic supplementary material:**

The online version of this article (doi:10.1186/s12915-015-0220-7) contains supplementary material, which is available to authorized users.

## Background

RNA binding proteins (RBPs) are multifunctional molecules that coordinate key functions such as pre-mRNA splicing, 5’ capping, 3’ end processing, nuclear-cytoplasmic transport, mRNA translation and storage, and mRNA processing [1, 2]. The scaffold attachment factor protein family consists of SAFB1, SAFB2 and a RBP termed SAF-like transcription modulator (SLTM) [3]. They are large multi-domain proteins encoding a SAF box (DNA binding domain), an RNA recognition motif and Glu/Arg regions that mediate protein-protein interactions [3, 4]. Scaffold Attachment Factor B1 (SAFB1) is an evolutionarily conserved nuclear protein that has been associated with multiple cellular processes such as transcription, chromatin structure and apoptosis [4, 5]. Studies point to a role for SAFB1 in RNA processing and mass-spectrometric analyses identified SAFB1 amongst approximately 800 proteins associated with mRNA [6, 7]. SAFB1 was first identified attached to the scaffold/matrix attachment regions of chromatin [8]. It was subsequently identified as HET/SAFB1, a protein that downregulates the transcription of the hsp27 gene [9] and hnRNPA1 associated protein, a factor recruited to nuclear stress bodies [10]. Studies of SAFB1 function have shown scaffold/matrix attachment regions serve as anchors to attach chromatin to the nuclear matrix [8] and the chromatin loop domains formed at these attachment regions are thought to be sites controlling transcription and replication [11, 12]. SAFB1 has also been shown to interact with proteins involved in splicing and other aspects of RNA-processing, such as AUF1/hnRNP D, hnRNP A1, SRSF1 (ASF/SF2), SRSF7 [5, 13–16], SRrp86 [17], SLM-1 [18], T-STAR, and Sam68 [19]. The interaction of SAFB1 with regulators of RNA processing [20], together with its high affinity for RNA polymerase II [12], provides further evidence for its involvement in the assembly of RNA regulatory complexes and alternative splicing [21, 22]. SAFB1 knockdown was shown to regulate splice site selection of the tra2β1 gene, further supporting a role in alternative splicing [19, 23]. SAFB1 is ubiquitously expressed in most tissues and is expressed at high levels in the developing and mature brain [13]. Following heat stress, SAFB1 translocates to nuclear stress bodies together with HSF1 and Sam68. It has been hypothesised that nuclear stress bodies may sequester splicing factors to downregulate normal cellular splicing and facilitate the processing of RNA transcripts that are essential for the stress response [10, 24].

Neurons transcribe a greater number of genes than other cell types [25] and hence RBPs must be expressed at high levels to coordinate the processing of RNA [26]. Evidence also suggests that aging alters the expression and function of some RBPs and this contributes to the decline in cognition and increased susceptibility to specific neurological conditions [27, 28]. SAFB1 is expressed at high levels in neurons and its expression is regulated by stress, suggesting it may play an important role in coordinating the expression of neuroprotective proteins. Surprisingly, although considerable evidence suggests SAFB1 regulates RNA processing, its specific interactions with RNA have not been studied. To investigate the functional role of SAFB1 and to identify interactions with individual RNAs in neuronal cells, we used individual-nucleotide resolution cross-linking and immunoprecipitation with deep sequencing (iCLIP-Seq) [29, 30]. We found SAFB1 cross-link sites were highly enriched in exons and the purine-rich pentamers, GAAGA and AAGAA, are the most significantly enriched motifs within the binding sites. Further analyses using high-resolution exon junction arrays showed there were significant changes in the splicing patterns of a number of genes following SAFB1 knockdown. In addition, we analysed the frequency of predicted SAFB1 target motifs [31] and found that there was a significant increase in the occurrence of paired trimer motifs within the last 50 nt of exons downregulated following SAFB1 knockdown. The results also showed that SAFB1 regulated splicing within an NCAM1 minigene but this regulation was lost when the AGA, GAA, AAG recognition motifs were mutated. NCAM1 and many RNAs bound by SAFB1 have roles in neuronal plasticity and function. Furthermore, empirical analyses showed that SAFB1 regulated dendritic spine size in hippocampal neurons, confirming that SAFB1 plays an important role in coordinating gene processing in neurons.

## Results

### SAFB1 iCLIP analyses

**Fig. 1:**
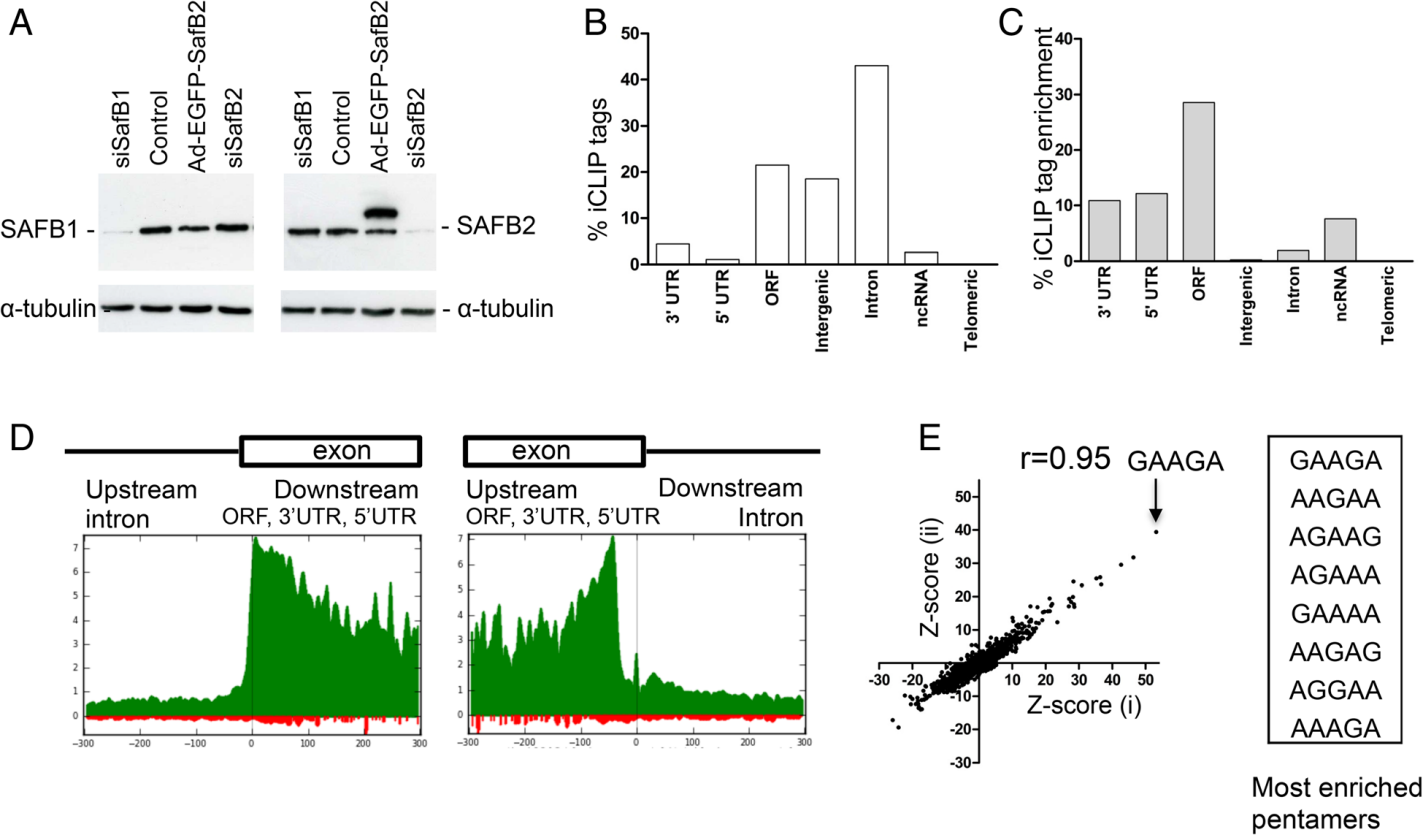
Analyses of SAFB1 RNA-interactions by iCLIP. **a** Western blots showing that following siRNA-mediated knockdown of SAFB1 and SAFB2, the SAFB1 Ab detects a reduction in endogenous SAFB1 but not endogenous SAFB2 and neither does the Ab recognize SAFB2 when mediated by an adenovirus expressing EGFP-tagged SAFB2. Conversely, the SAFB2 antibody (second blot) shows a reduction in endogenous SAFB2 expression following treatment with the SAFB2 siRNA and also detects the EGFP tagged SAFB2 protein. **b** The bar graphs show the proportion of cDNAs from the iCLIP experiments and their mapping to different genomic regions (human genome hg19) (**c**) and normalized iCLIP read densities (iCLIP-tags divided by average length of region) mapped to different genomic regions. **d** SAFB1 crosslink sites mapped around the 3’ and 5’ splice sites from −300 to +300 nucleotides with the splice junction at position 0. Data are normalized by all junctions spanning position, smoothed and represented as 10^9^ × [number of crosslinks]/[total number of crosslinks]. **e** Z-scores of pentamer occurrence within the 61-nt sequence surrounding crosslink sites calculated by plotting data from individual iCLIP experiments (detailed in the results section). The most enriched pentamers are shown and SAFB1 recognized the GAAGA pentamer with the highest frequency

The sequences of SAFB1 and SAFB2 share considerable identity and we therefore assessed the specificity of the anti-SAFB1 antibody. The results (Fig. 1a) showed that the SAFB1 antibody detected a reduction in SAFB1 expression following the siRNA-mediated knockdown of SAFB1 but not following the knockdown of SAFB2. In addition, the SAFB1 antibody did not detect enhanced green fluorescence protein (EGFP)-tagged SAFB2 when expressed exogenously by an adenovirus (Fig. 1a). Following confirmation of antibody-specificity, iCLIP experiments were carried out in triplicate on SH-SY5Y neuroblastoma cells. Cells were UV-crosslinked to mediate the formation of protein-RNA complexes and the protocol validated using cell extracts from non- crosslinked cultures (no UV) and those transduced with an adenoviral vector expressing SAFB1. In the absence of UV crosslinking, ^32^P-end-labelled RNA was not immunoprecipitated with SAFB1 (Additional file 1). iCLIP was performed with partial RNase I digestion and, following SDS-PAGE analysis of immunoprecipitated SAFB1, the cross-linked RNA was extracted, reverse-transcribed and PCR-amplified before being subjected to high-throughput sequencing using Illumina GA2. Removing sequences that truncated at the same nucleotide in the genome and shared the same random barcode eliminated PCR amplification artefacts. This left a total of 0.4 million unique sequence reads that aligned to the human genome hg19, allowing only a single nucleotide mismatch, where each read represents a single cDNA molecule. In the no antibody control, ~30,000 (0.08 % of the 0.4 million SAFB1 reads) reads were found. The distribution of the iCLIP-tags to RNA was analysed (as a percentage of the total number of tags) and mapped to open reading frames, intergenic and intronic regions, non-coding RNA (ncRNA), and untranslated regions (UTRs) (Fig. 1b). We also mapped the fold enrichment of CLIP-tag density (the number of CLIP-tags divided by the length of each RNA feature) in different RNAs relative to the average CLIP-tag density in the genome (Fig. 1c). The tags were highly enriched in open reading frames (30-fold), 3’ and 5’ UTRs (10-fold), and non-coding RNAs. Within genes, the highest densities of SAFB1 cross-link sites (iCLIP tags divided by average length of region) were located in exons in proximity to intron-exon and exon-intron boundaries (Fig. 1d). SAFB1 cross-link sites fall predominantly within exons, suggesting a role in pre-mRNA splicing or in the fate of mRNPs after splicing. To investigate the RNA sequence specificity of SAFB1, the Z-scores of possible pentamers within the 61-nucleotide sequence surrounding all cross-link sites (−30 nt to +30 nt) were calculated by plotting the experimental repeats against each other (i.e. repeat 1 vs. repeat 2, repeat 2 vs. repeat 3, and repeat 1 vs. repeat 3). In each case, the GAAGA motif was found to be the most significantly enriched (Additional file 2). We also included the Z-scores (Additional file 2) obtained from iCLIP experiments investigating the interaction of SAFB1 under stress conditions. When these data were analysed together, the most significantly enriched pentamers were again GAAGA and AAGAA (Pearson coefficient r = 0.95) (Fig. 1e). These data also identify the GAA, AAG and AGA trimers as the core motifs likely to be recognised by SAFB1 [31]. To assess the potential functions of SAFB1, a gene ontology (GO) analysis of the RNAs with greater than 5 tags (Additional file 3) was carried out and the most significant biological processes are shown (Table 1). SAFB1 was predicted to be involved in the expression of proteins involved in chromosome organisation, RNA processing (e.g. splicing, processing, transport), the cellular response to stress, and neuron projection and morphogenesis.

**Table 1:** Significant gene ontology (GO) terms from the iCLIP data for genes with >5 tags

Category	GO term	Description	Fisher Exact/EASE Score
Biological Process	GO:0007049	Cell cycle	4.3 × 10^–21^
Biological Process	GO:0051276	Chromosome organization	1.4 × 10^–20^
Biological Process	GO:0022402	Cell cycle process	2.5 × 10^–19^
Biological Process	GO:0006259	DNA metabolic process	1.5 × 10^–18^
Biological Process	GO:0006396	RNA processing	3.5 × 10^–18^
Biological Process	GO:0006325	Chromatin organization	3.5 × 10^–16^
Biological Process	GO:0016071	mRNA metabolic process	6.2 × 10^–16^
Biological Process	GO:0006974	Response to DNA damage stimulus	1.8 × 10^–15^
Biological Process	GO:0006397	mRNA processing	3.9 × 10^–15^
Biological Process	GO:0016568	Chromatin modification	4.9 × 10^–15^
Biological Process	GO:0000279	M phase	1.3 × 10^–14^
Biological Process	GO:0008380	RNA splicing	1.7 × 10^–14^
Biological Process	GO:0006281	DNA repair	3.2 × 10^–14^
Biological Process	GO:0006260	DNA replication	7.7 × 10^–14^
Biological Process	GO:0033554	Cellular response to stress	4.9 × 10^–13^
Biological Process	GO:0046907	Intracellular transport	5.6 × 10^–13^
Cellular Component	GO:0031981	Nuclear lumen	8.1 × 10^–66^
Cellular Component	GO:0070013	Intracellular organelle lumen	1.3 × 10^–59^
Cellular Component	GO:0043228	Non-membrane-bound organelle	6.1 × 10^–58^
Cellular Component	GO:0043233	Organelle lumen	9.6 × 10^–58^
Cellular Component	GO:0031974	Membrane-enclosed lumen	2.4 × 10^–57^
Molecular Function	GO:0005524	ATP binding	1.3 × 10^–27^
Molecular Function	GO:0003723	RNA binding	7.9 × 10^–27^
Molecular Function	GO:0001882	Nucleoside binding	1.2 × 10^–24^
Molecular Function	GO:0001883	Purine nucleoside binding	6.4 × 10^–24^
Molecular Function	GO:0000166	Nucleotide binding	1.4 × 10^–23^
Molecular Function	GO:0004386	Helicase activity	1.7 × 10^–19^

**Fig. 2:**
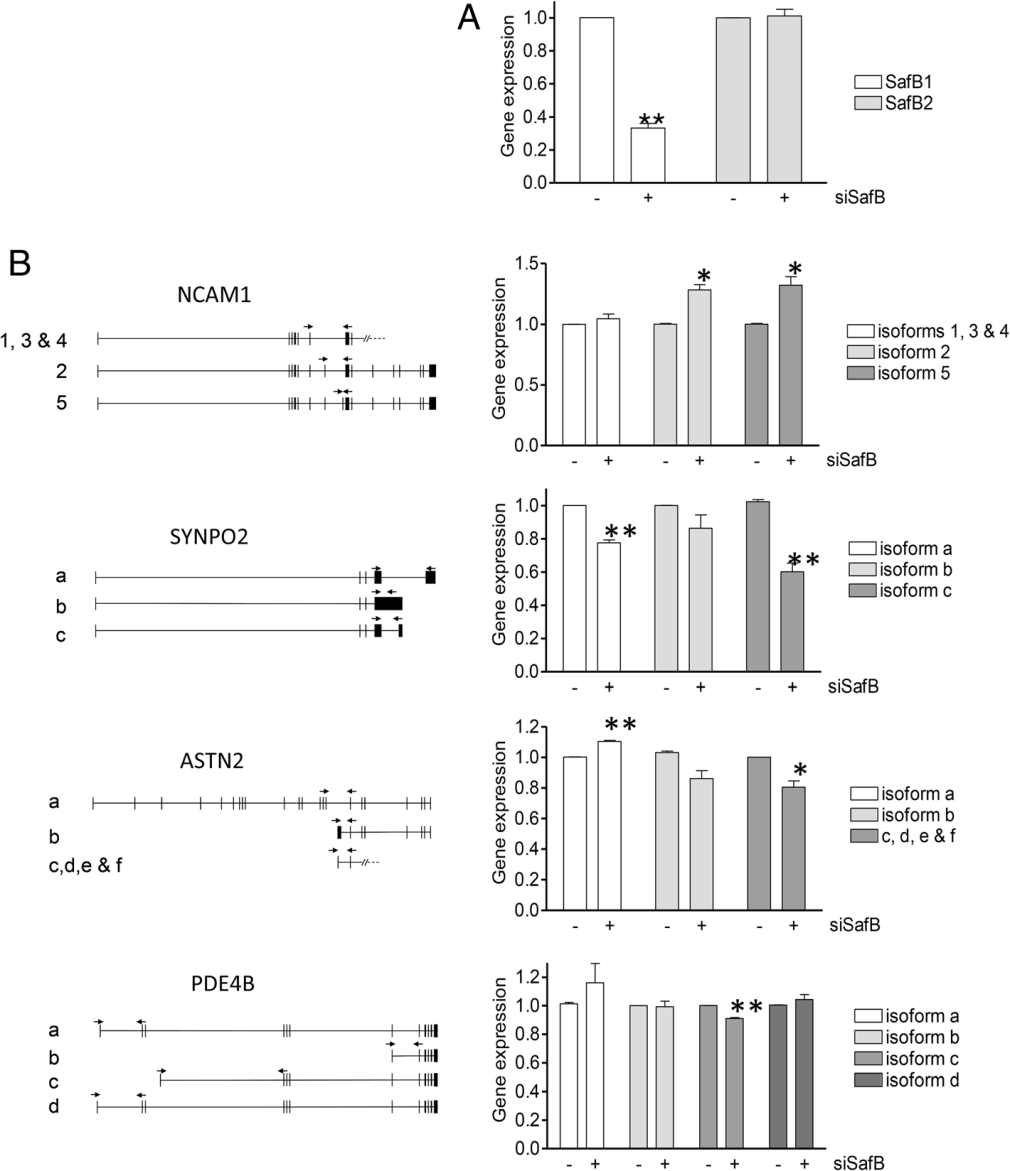
SAFB1 knockdown mediates changes in the expression of specific RNA isoforms. RT-PCR analysis of genes with crosslinks identified by iCLIP was performed in such a way as to examine the possibility of transcript-specific alterations. **a** RNA was isolated from SHSY5Y cells following siRNA mediated SAFB1 knockdown and SAFB1 and SAFB2 RNA levels measured. **b** Specific primer sets were designed and used to measure the expression of NCAM1, SYNPO2, ASTN2 and PDE4B isoforms. The results were obtained following three independent experiments. Statistical analysis was by ANOVA followed by post-hoc *t*-tests. *P <0.05, **P <0.01

**Fig. 3:**
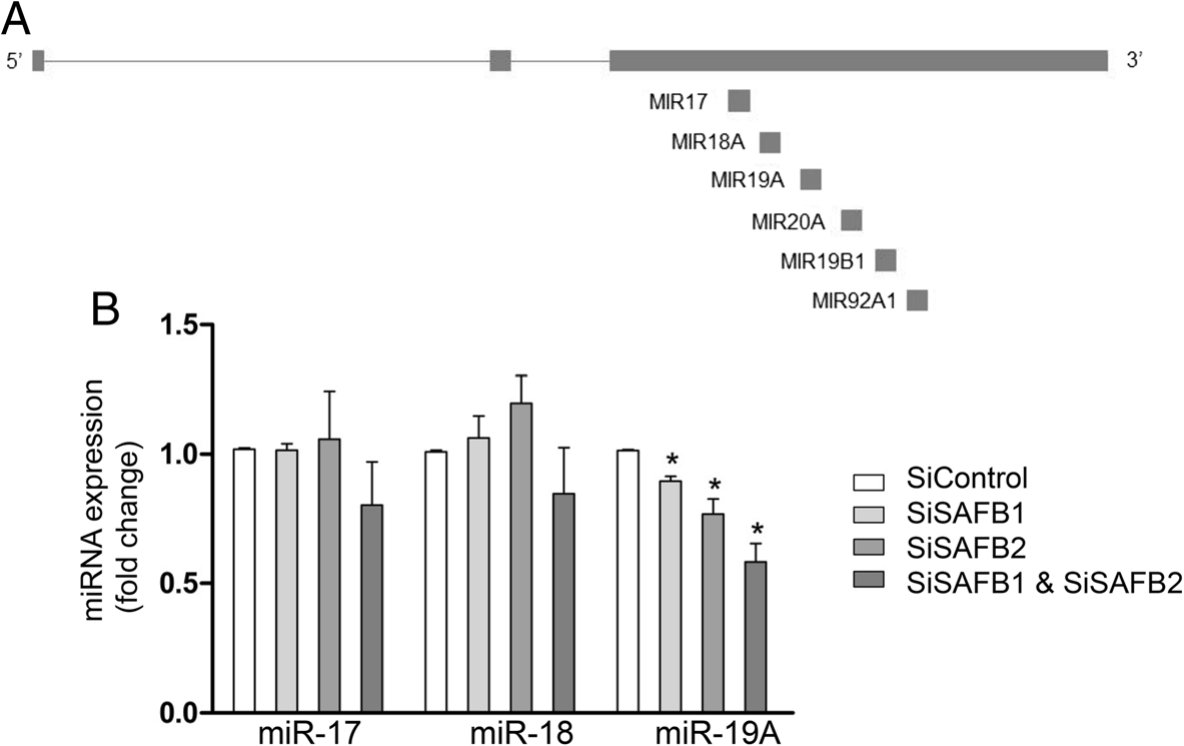
SAFB1 regulates miR17HG. **a** Schematic representation of the miRNA encoded in the miR17HG (miR17-92 cluster). **b** Knockdown of SAFB1 mediates a decrease in the level of miR-19A. Means ± SD are shown. ANOVA followed by post-hoc *t*-tests. *P <0.05

Real-time PCR analysis of functionally-related genes of interest (NCAM1, SYNPO2 (both alternatively spliced), and ASTN2 and PDE4B (alternative promoters)) with iCLIP crosslinks was performed to investigate a functional interaction. Three of these genes (ASTN2, PDE4B and SYNPO2) contained greater than 100 tags (total count), while NCAM1 contained 20. Total RNA was isolated from SH-SY5Y cells following siRNA-mediated SAFB1 knockdown (Fig. 2a), reverse-transcribed and PCR-amplified (Fig. 2b). Several specific regulatory changes were identified in this way, for instance, SAFB1 knockdown significantly altered the level of specific NCAM1 and SYNPO2 isoforms. These results are suggestive of an effect of SAFB1 on alternative splicing since different splicing patterns are used to generate these isoforms. iCLIP tags found in tra2β1 further support SAFB1’s role in alternative splicing, as splice site selection for this gene is regulated by SAFB1 [19, 23]. The expression of ASTN2 and PDE4B was also altered by SAFB1 knockdown with increased and decreased isoform expression being noted likely due to effects of SAFB1 on alternative promoters. Furthermore, the expression of genes found to have a high number of SAFB1 tags (e.g. MAP3K7, 255 tags) were also found to be significantly altered following SAFB1 knockdown (unpublished observations, Idris, J). SAFB1 tags were also found in the genes of non-coding RNAs so the effect of SAFB1 (and for comparison SAFB2) knockdown on the processing of mature miRNAs from the MIR17HG (miR-17-92) miRNA cluster was analysed further. We found that both SAFB1 and SAFB2 knockdown mediated a decrease in the expression/processing of miR19a, but no effect on miR-17 or miR-18 was noted (Fig. 3). The simultaneous knockdown of SAFB1 and SAFB2 also mediated a significant decrease in the expression of miR-19A. In addition, RNAseq experiments showed SAFB1 knockdown significantly increased expression of the primary miR-17-92 transcript (unpublished observations, Rivers C). Taken together, these results validate the iCLIP results and show that SAFB1 is interacting with specific coding RNA transcripts to modulate transcript expression and possibly regulate alternative splicing.

### Investigating the regulation of alternative splicing by SAFB1 using NCAM1 minigenes

**Fig. 4:**
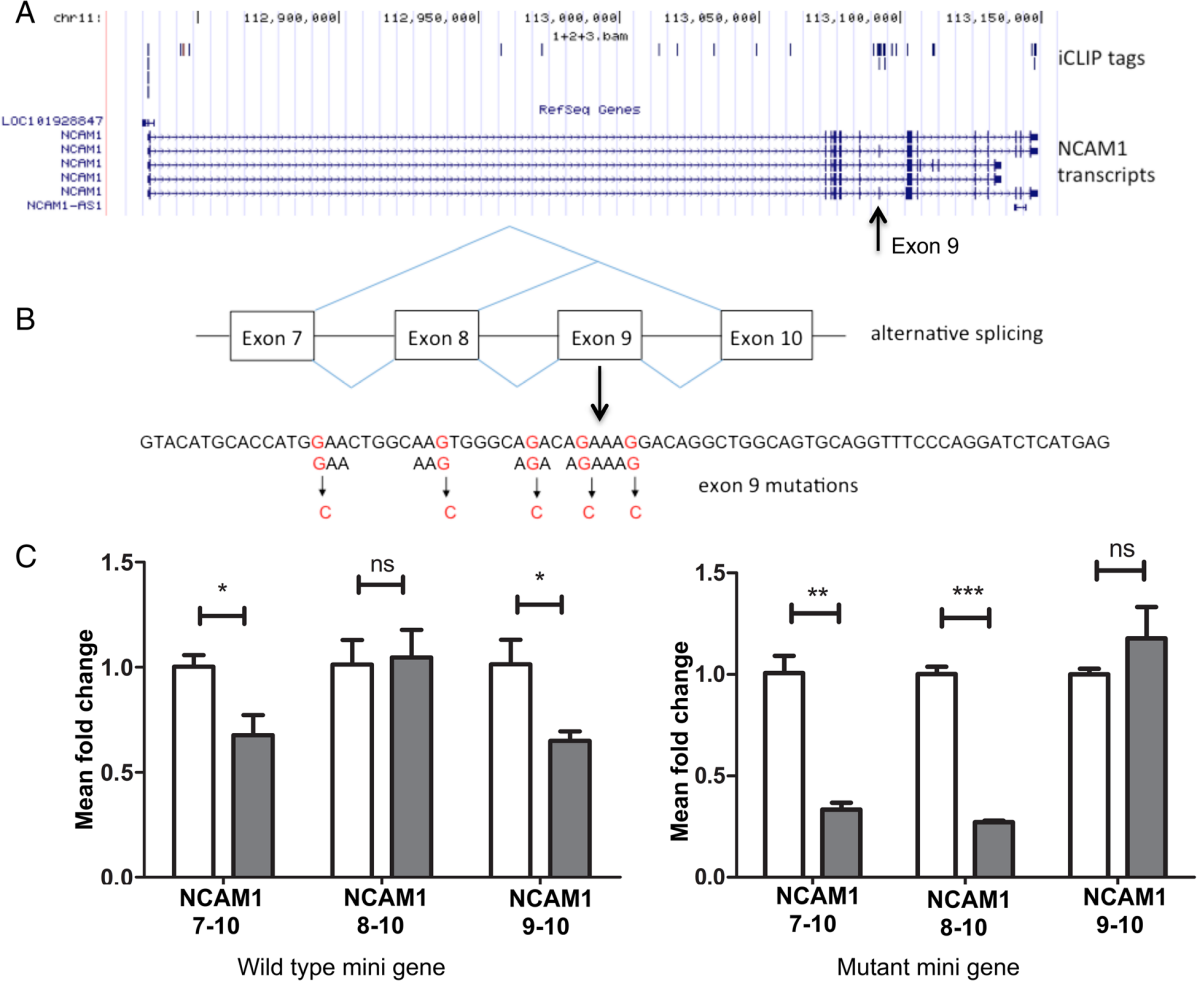
SAFB1 regulates splicing from wild type and mutant NCAM1 minigenes. **a** The distribution of SAFB1 iCLIP tags within the NCAM1 minigene is shown and an arrow indicates the position of exon 9 in transcript variant 5. **b** The schematic indicates the composition of the minigenes and splice forms generated. The sequence of wild type exon 9, the location of the GAA/AAG and AGA motifs, and the mutations introduced to alter all AAG/AGA/GAA are shown. **c** PCR analyses using exon-specific spanning primers was used to analyse the effect of SAFB1 knockdown on the expression of the NCAM1 isoforms (NCAM1 7–10, 8–10 and 9–10) in the wild type and mutant minigenes. The open bars represent expression of the transcript indicated with non-targeting control knockdown and the closed bars following SAFB1 knockdown. Values are means ± SEM, statistical analysis was performed by ANOVA and post-hoc *t*-tests *P <0.05, **P <0.01, ***P <0.001

The iCLIP analysis suggested that SAFB1 may play a role in alternative splicing; to investigate this further, we used a NCAM1 minigene. We chose to use NCAM1 as (1) both SAFB1 and NCAM1 are highly-expressed in the hippocampus and a significant body of work has shown that NCAM1 is an important regulator of synaptic function [32], and its altered expression [33] and splicing [34] is implicated in the aetiology of human neuropsychiatric illnesses; and (2) since SAFB1 iCLIP tags were enriched over exon 9 (Fig. 4a), it was appropriate to construct a wild type NCAM1 minigene with exons 7, 8, 9 and 10 (Fig. 4b) and a mutant minigene in which mutations were introduced to alter all AAG/AGA/GAA trimers in exon 9 (Fig. 4b). Using RT-PCR and qPCR analyses (n = 3) we found that expression of the NCAM1 9–10 isoform was significantly reduced following SAFB1 knockdown (Fig. 4c). This effect was abolished when trimers were mutated in exon 9, supporting the notion that direct binding of SAFB1 to exon 9 enhances expression of the 9–10 isoform from the minigene (Fig. 4c). Regulation of expression of the NCAM 7–10 and 8–10 isoforms appears to be somewhat more complicated, with mutation of trimers in exon 9 making expression of these isomers more dependent on SAFB1. The latter results suggest that SAFB1 might be enhancing expression of the 7–10 and 8–10 isoforms by binding elsewhere in the minigene, or perhaps via an indirect effect.

### Exon microarray analysis

**Fig. 5:**
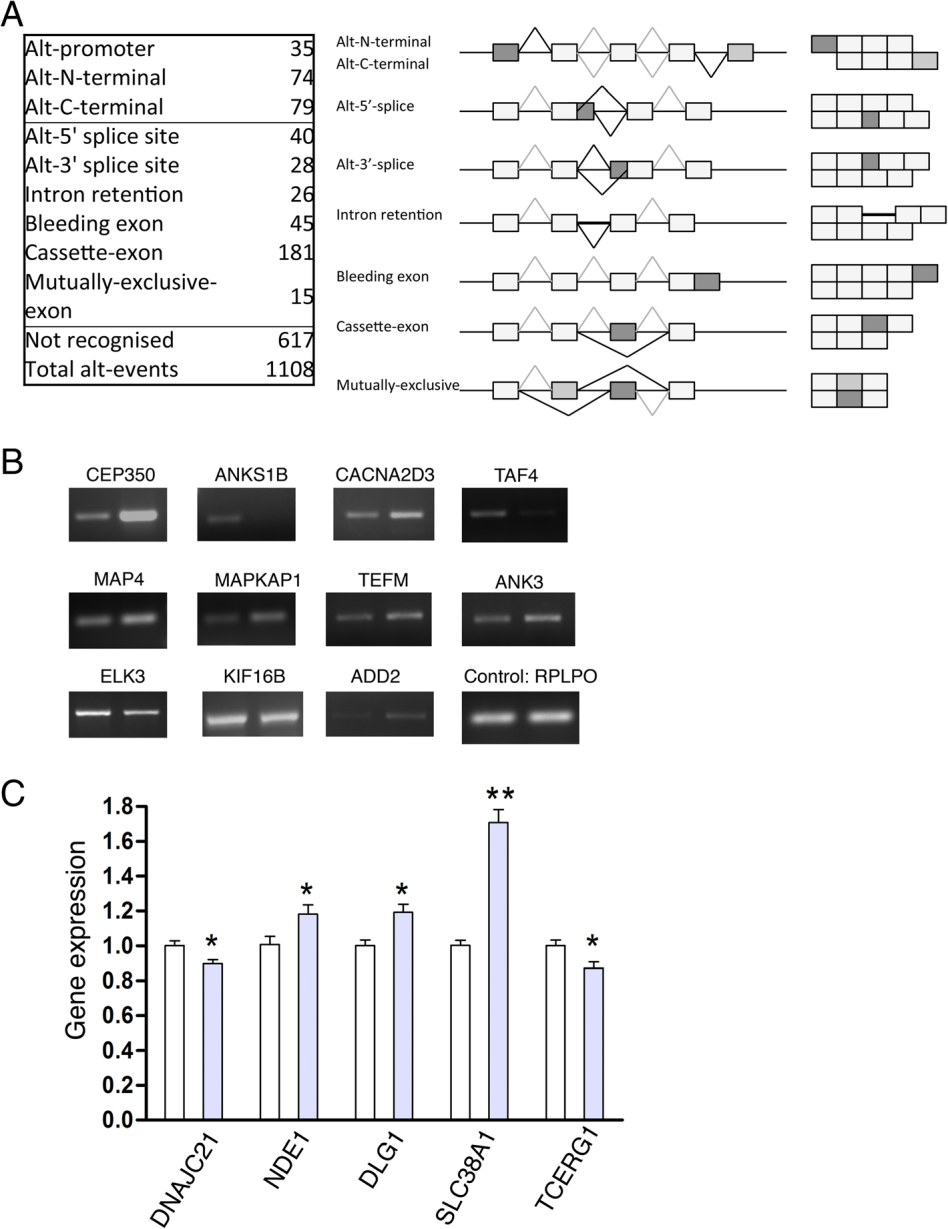
Exon array analyses following SAFB1 knockdown. Samples were prepared from SYSY5Y cells following siRNA-mediated SAFB1 knockdown and 1.0 ST Affymetrix high-resolution exon arrays used to analyse effects on splicing. AltAnalyse was used to interrogate the microarray data for alternative splicing events and the significant changes are shown (**a**). RT-PCR was used to confirm the splicing events identified by arrays (**b** and **c**). Primer pairs were generated to distinguish isoforms and three independent experiments were carried out in triplicate. SAFB1 knockdown mediated statistically significant changes in expression of the gene transcripts shown in (b and c). Statistical analysis was by ANOVA followed by post-hoc *t*-tests. **P <0.01, *P <0.05. B lane 1 control; lane 2 SAFB1 knockdown. Open bars in (c) represent controls, filled bars represent SAFB1 knockdown

To further investigate the association of SAFB1 with splicing, microarray analysis with a human exon 1.0 ST Affymetrix array was used (Fig. 5). Triplicate samples were prepared from SYSY5Y cells following siRNA-mediated SAFB1 knockdown and transfection with a siRNA non-targeting control. We used AltAnalyse (an alternative splicing tool) [35] to interrogate the microarray data for alternative exon expression. Following SAFB1 knockdown, we found 79 exons were significantly downregulated and 87 exons were upregulated (MIDAS values <0.05 with >2-fold changes). Summaries of the significant positive and negative changes found by AltAnalyse are shown in Fig. 5a, which show that SAFB1 can promote and silence expression of RNA elements. Next, we confirmed these results using RT-PCR to assess 23 splicing events identified by arrays. Primer pairs were generated to distinguish isoforms, and 18 of these reproduced the direction of the observed splicing change, resulting in a 78 % (18/23) validation rate of the microarray data (Fig. 5b,c). We also investigated the effects of SAFB1 on non-coding transcripts. SAFB1 was found to have a very high number of cross-link sites across the long non-coding RNA (lncRNA) MALAT1 but not in the area that encodes the cleaved MASC1 transcript. Further experiments using MALAT1-specific PCR showed that SAFB1 knockdown significantly increased the expression of MALAT1 (Additional file 4). We also conducted a GO analysis of genes that the exon array identified as being differentially expressed (altered by >2-fold and *P* <0.01) following SAFB knockdown (Table 2). This analysis linked SAFB1 interactions with the expression of genes associated with meiosis, the cytoskeleton, and/or involved with ATP/nucleoside binding, cytoskeletal organisation and microtubule-based processes.

**Table 2:** Enrichment of gene ontology (GO) terms in genes altered >2.0-fold by SafB1 knockdown identified by exon array analysis

Category	GO term	Description	Fisher Exact/EASE Score
Biological Process	GO:0051327	M phase of meiotic cell cycle	9.9 × 10^–6^
Biological Process	GO:0051321	Meiosis	9.9 × 10^–6^
Biological Process	GO:0051321	Meiotic cell cycle	1.3 × 10^–5^
Biological Process	GO:0000279	M phase	1.7 × 10^–5^
Biological Process	GO:0032886	Regulation of microtubule-based process	2.0 × 10^–4^
Biological Process	GO:0022403	Cell cycle phase	5.3 × 10^–4^
Biological Process	GO:0007494	Midgut development	6.4 × 10^–4^
Biological Process	GO:0007017	Microtubule-based process	7.6 × 10^–4^
Biological Process	GO:0000226	Microtubule cytoskeleton organization	8.8 × 10^–4^
Cellular Component	GO:0012505	Endomembrane system	6.3 × 10^–5^
Cellular Component	GO:0015630	Microtubule cytoskeleton	3.5 × 10^–4^
Cellular Component	GO:0005874	Microtubule	5.7 × 10^–4^
Cellular Component	GO:0005856	Cytoskeleton	1.1 × 10^–3^
Molecular Function	GO:0001882	Nucleoside binding	3.2 × 10^–6^
Molecular Function	GO:0005524	ATP binding	3.3 × 10^–6^
Molecular Function	GO:0032559	Adenyl ribonucleotide binding	3.4 × 10^–6^
Molecular Function	GO:0030554	Adenyl nucleotide binding	3.7 × 10^–6^
Molecular Function	GO:0001883	Purine nucleoside binding	4.1 × 10^–6^
Molecular Function	GO:0032553	Ribonucleotide binding	1.7 × 10^–5^
Molecular Function	GO:0032555	Purine ribonucleotide binding	1.7 × 10^–5^
Molecular Function	GO:0000166	Nucleotide binding	1.7 × 10^–5^

### Distribution of motifs recognised by SAFB1 using RNAmotifs

**Fig. 6:**
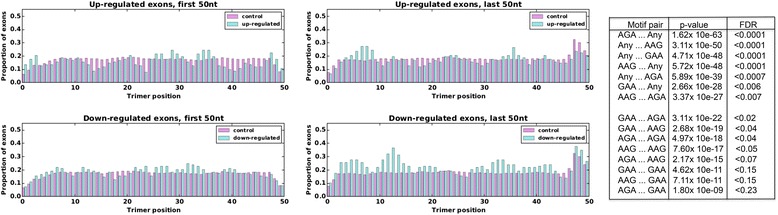
Distribution of differentially expressed purine-rich trimer exons following SAFB1 knockdown. Bar graphs showing the proportion of exons that contain the trimers of interest at particular locations in upregulated (top) and downregulated (bottom) exons compared to controls. The table shows the most significant motif pairs found in the last 50 nt of downregulated alternative exons for the three trimers (AAG, AGA and GAA) along with degenerate pairs defined as a specific target trimer paired with AAG, AAG or AGA (Any). Shown are the most highly significant pairings over-represented in downregulated exons, (from most to least significant). Bootstrapping with 10,000 samples yielded the estimated false discovery rates

Recently, the clustering property of specific elements/motifs (recognised by RNA binding proteins) motivated the development of the RNAmotifs software that identifies multivalent motifs predicted to regulate splicing [31]. This method assumes most RNA-binding domains recognise a core of up to four nucleotides and our iCLIP analyses suggest the GAA/AAG/AGA nucleotide sequences form the core motifs recognised by SAFB1. As the core motifs were already predicted by iCLIP analyses, we modified the RNAmotifs approach to analyse the distribution of these (AAG/AGA/GAA) trimers within exons differentially expressed following SAFB1 knockdown (Fig. 6 and details of the analysis are given in the methods section). We initially looked at the proportion of differentially expressed exons (following SAFB1 knockdown) that contained the trimers of interest at particular locations in upregulated and downregulated exons compared to our control exons. In the first 50 nt of differentially expressed upregulated and downregulated exons we did note differences compared to controls. However, there was an enrichment of the SAFB1 trimer motifs in the last 50 nt of downregulated cassette exons, indicating that there may be increased inclusion of the cassette exon in the presence of SAFB1. To obtain an approximate false discovery rate (FDR) for these hits, we used a bootstrapping approach that selected exons randomly from the genome, counted pair motifs in each exon, and then compared the counts with our control set. In each trial we sampled 109 exons without replacement from a population of 54,263 known exons and used Fisher’s exact test to compare the frequencies of each motif pair with the frequencies in the control set. The FDR was then given by bootstrapping 10,000 samples and *P* values greater than 0.0005 were considered not significant (Fig. 6). By far the most significant pair motif was AGA paired with any of the three trimers (AAG, GAA, AGA) found downstream of it (*P* = 1.6 × 10^−63^, FDR below 0:0001). This result is consistent with those made by Cereda et al. [31], in that it suggests RBPs bind multivalent patterns to modulate splicing.

### SAFB1 expression and function in neurons

**Fig. 7:**
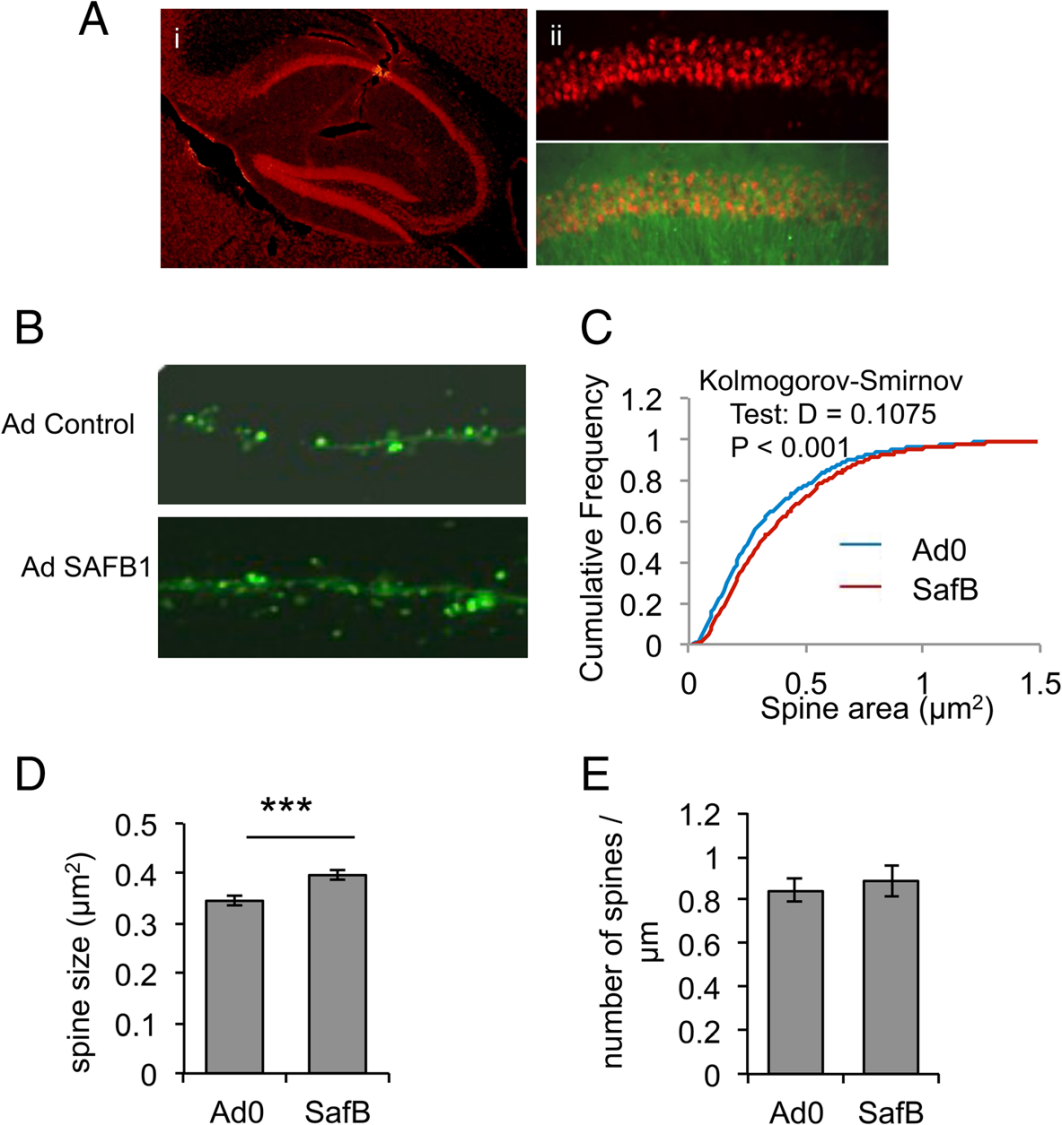
SAFB1 regulated dendritic spine size in primary hippocampal neurons. **a** Immunocytochemical analyses show SAFB1 is highly expressed in the hippocampus (i). SAFB1 is clearly evident in pyramidal neuron cell bodies in the CA1 region of the hippocampus ((i) red SAFB1 immunofluorescence) and a neurofilament stain of dendrites (green immunofluorescence (ii) second panel) shows SAFB1 is located exclusively in neuronal cell bodies. Primary hippocampal neurons were transduced with SAFB-EGFP or EGFP alone (confocal images shown in (**b**) and confocal microscopy used to measure spine (**c**) area, (**d**) size, and (**e**) density. Spine size measures were analysed by cumulative frequency using the Kolmogorov-Smirnov test (b, d = 0.1075; *P* <0.001) and by *t*-test (c) ***P <0.001. Values shown in bar graphs are means ± SD

The NCAM1 minigene experiments together with the iCLIP and exon array analyses suggest SAFB1 may play an important role in regulating the organisation of the cytoskeleton, neuronal development and synaptic function. Immunocytochemical analyses showed that SAFB1 expression was evident throughout the brain but was most pronounced in hippocampal neurons (Fig. 7a). Therefore, to investigate effects of SAFB1 on indices of synaptic function we transduced primary hippocampal neurons that had been 14 days in culture (to develop a mature dendritic network) with an adenovirus expressing SAFB1. Four days later, we fixed the cells and used confocal microscopy to analyse spine number and size (Fig. 7b). The results showed that SAFB1 expression significantly increased spine size (*P* <0.001) but had no effect on spine number (Fig. 7c).

## Discussion

Following transcription, RNA transcripts immediately associate with proteins that facilitate their processing and export. SAFB1 (like hnRNPs and SR proteins) can bind RNA via its N-terminal RNA recognition motifs; however, these interactions have not yet been investigated using genomic profiling techniques. We used the powerful iCLIP-seq method to identify RNA targets of SAFB1 and our results showed it interacted with hundreds of RNA species with the greatest enrichment being in exons, non-coding RNAs, and 5’ and 3’ UTRs. SAFB1 binding to exons was associated with the purine-rich pentamers, GAAGA and AAGAA, that occurred with the highest frequency in proximity to intron-exon and exon-intron boundaries. GO analyses based on these interactions suggested SAFB1 was likely to have roles in regulating expression of proteins involved in RNA processing (splicing, processing, transport), the cellular response to stress, and neuron projection and morphogenesis. NCAM1, SYNPO2, ASTN and PDE4B are examples of genes with multiple SAFB1 tags and further investigation, including NCAM1 minigene experiments (also discussed below), confirmed SAFB1 knockdown did alter their expression. Interestingly, NCAM1 [36], ASTN2 [37], and PDE4B [38] are known to play important roles in regulating synaptic function and are implicated in human psychiatric disease. Importantly, SAFB1 was also found to regulate the processing of non-coding RNAs. SAFB1 has multiple cross-link sites along the lncRNA MALAT1 and SAFB1 knockdown increased MALAT1 expression. MALAT1 has been shown to interact with multiple SR proteins (dictating their location and phosphorylation state) to regulate alternative splicing. Previous studies using iCLIP have shown that the splice regulators TDP-43, SRSF, SRSF3 and SRSF4 also cross-link extensively with MALAT1 [39–41] and this interaction may therefore be common with RBPs that regulate splicing. iCLIP-seq analyses also showed that SAFB1 interacted with the physiologically important miR-17-92 miRNA cluster [42]. We further showed that this interaction had functional consequences, with SAFB1 knockdown being found to significantly increase the expression of the miR-17-92 primary transcript and reduce the expression of miR-19a. Interestingly, and related to the functions predicted by the GO analyses, the miR-17-92 cluster (and miR-19a specifically) has been shown to be an important regulator of axonal function [42]. Furthermore, miR-17-92 cluster dysregulation has been implicated in a number of cancers [43], human aging and in neurodegenerative disease [44].

In this study, we validated interactions with genes that had a high and comparatively low number of SAFB1 tags suggesting the presence of iCLIP tags was a good predictor of functionally important interactions. To further investigate the role SAFB1 plays in alternative splicing we used RNAmotifs to analyse the enrichment and distribution of the GAA, AAG and AGA binding motifs in differentially expressed exons. This analysis allowed position-dependent effects on exon inclusion/exclusion following SAFB1 binding to be predicted. The frequency of one or more motif pairs (for example, AGA followed by AGA, GAA or AAG) was significantly increased within downregulated exons suggesting SAFB1 in these cases promoted exon inclusion. SAFB1 iCLIP tags were found in the NCAM1 gene and NCAM1 minigene experiments showed that SAFB1 knockdown altered alternative splicing and that mutation of the GAA, AAG and AGA motifs significantly decreased this regulation. These data therefore support the ICLIP and RNAmotifs analyses, which together suggested SAFB1 regulates the alternative splicing of specific genes via recognition of AG rich motifs. SAFB1 has been implicated in the regulation of a number of cellular processes; however, as it is a multi-domain protein defining specific roles has proved difficult. The regulation of NCAM1 splicing and its interaction with known splicing regulators and with polymerase II suggests it may be involved in spliceosome assembly. We provide evidence supporting this hypothesis and suggest that SAFB1 may act to recruit splicing factors to a small number of pre-mRNAs. The DEAD-box helicase eIF4AIII, a core component of the exon junction complex (EJC) was found to bind within exon regions rich in GAAGA motifs at sites distal and close to EJC regions [45]. As well as SAFB1 and eIF4AIII, a number of SR proteins have been found to bind the (GAA)n motif, including SRSF1 (SF2/ASF), hTra2 and SRm160 [46]. It was suggested that GAAGA might be a high-affinity binding site for factors that influence EJC binding; as such, it is possible that these proteins bind this motif to attract EJC factors during splicing and/or to stabilize the EJC in specific genes. SAFB1 expression levels differ between tissues and, as its expression levels in (non-dividing) neurons (e.g. hippocampal and cerebellar) are high, it is therefore possible that SAFB1 plays a more significant role in regulating RNA processing in these tissues. The level and sub-cellular location of SAFB1 also differs following stress and this may also influence the degree to which SAFB1 competes with other (SR) proteins for the recognition site. Similarly, as SAFB1 is known to bind other splice regulators, its expression levels may also influence the sequence preference/specificity of spliceosome complexes and thereby regulate cellular function.

GO functional analyses (of genes with differentially expressed exons) suggested SAFB1 regulates the expression of proteins involved in ATP/nucleoside binding, cytoskeletal organisation and microtubule-based processes. Importantly, minigene experiments showed the alternative splicing of NCAM1 was altered by SAFB1. NCAM1 is an important regulator of synaptic function [32] and its altered expression [33] and splicing [34] are implicated in the aetiology of human neuropsychiatric illnesses. SAFB1 mediated isoform-specific changes in ANK3 [47], ANKS1B [48], SAP97 (DLG1) [49], ADD2 [50], KIF16B [51, 52], and ELK3 [53], as well as in genes linked to the cytoskeleton and/or synaptic function and the aetiology of neurologic disease. For example, the ankyrin-G protein encoded by Ank3 organizes synaptic microtubules and is involved in protein trafficking, neurogenesis and neuroprotection, and in the aetiology of bipolar disorder and schizophrenia [47]. SAFB1 is located at 19p13.3-p13.2 and separate studies have found SNPs or duplications at this locus are associated with increased susceptibility to schizophrenia [54–56]. These data (and those showing that SAFB1 regulates dendritic spine number) suggest that altered function of SAFB1 could result in changes in the expression of genes that regulate synaptic function, some of which are also susceptibility genes for schizophrenia.

## Conclusions

Our global analysis shows SAFB1 interacts with distinct classes of RNA indicating it has multiple functions with regard to RNA processing and the control of gene expression and function. Importantly, we confirmed it binds primarily within exons and that SAFB1’s altered expression regulates the alternative splicing of genes with important roles in neuronal function (confirmed by NCAM1 minigene analysis). iCLIP data and further analysis using RNAmotifs predicted that SAFB1 is likely to interact with specific purine-rich trimer sequences to promote exon inclusion. The SAFB family consists of SAFB1, SAFB2 and SLTM, proteins that share considerable identity (particularly within their functional domains) and are all highly but differentially expressed in the central nervous system. The SAFB proteins will therefore have novel and unexplored roles in regulating the processing of coding and non-coding pre-mRNAs in the brain and, as such, represent a new and important family of RNA regulators.

## Methods

### iCLIP analysis

The iCLIP protocol was performed as described by König et al. [57], with modifications, including adding a heat inactivation step after circularization and using an adapter with a 3’ dideoxycytosine to reduce non-specific products [58]. SHSY5Y neuroblastoma cells were irradiated once with 400 mJ/cm^2^ at 254 nm in a Stratalinker 1800 and SAFB1 was immunoprecipitated with protein G Dynabeads (Invitrogen) conjugated to rabbit anti-SafB1 antibody (Bethyl laboratories Inc., A300-811A). The region corresponding to 140–200 kDa complexes was excised from the membrane to isolate the RNA and high-throughput sequencing of iCLIP cDNA was performed using Illumina GA2 with three replicate experiments on one flow cell lane. The random bar-code sequences for the individual experiments were applied using the following RT primers: 5’-phosphate-NNXXXXNNNAGATCGGAAGAGCGTCGTGGATCCTGAACCGC-3’, where the unique experimental identifier (XXXX) was as follows: AACC, ACAC and AGGT for the anti-SafB1 antibody (Bethyl) experiments and CGAC for a no antibody control. The random barcodes were registered and PCR-duplicates were removed along with any reverse-primer sequences. The filtered iCLIP tag sequences were mapped to the human genome sequence (version hg19/GRCh37) allowing one mismatch using Bowtie version 1.0.1. Comparing cross-link nucleotide positions from three independent replicates assessed reproducibility, and allowed the Z-score analysis of enriched pentamers at cross-link nucleotides to be calculated [59].

### RNA sequence analysis

We first identified the pentamers most enriched in the region of −30 to +30 nucleotides around the crosslink sites in the iCLIP data. The positional enrichment of these sets of pentamers around the crosslink sites was then determined by comparing the occurrence at each pentamer around the true crosslink site with the occurrence at randomized crosslink positions. The positions of crosslink sites were randomised within the same CDS.

### Transfection

SHSY5Y cells were transfected with ON-TARGETplus Human siRNA pools for SafB1, SafB2, SLTM or non-targeting (L-005150-00, L-020373-01, L-014434-01 and D-001810-10, Thermo Scientific) using lipofectamine-2000 (Invitrogen) according to the manufacturer’s instructions.

### Western blotting

Total cell lysates were prepared using RIPA buffer and the protein concentration was determined using Lowry’s assay (Bio-RAD). Equal amounts of protein were loaded on 6–10 % sodium dodecyl sulfate-polyacrylamide gels and transfer to a nitrocellulose membrane. Anti-HET/SAFB1 (Upstate, 05–588) and SafB1 antibody (Bethyl Laboratories Inc., A300-811A) were used together with anti-rabbit or anti-mouse IgG peroxidase conjugate (Amersham-Pharmacia) before visualization by enhanced chemiluminescence (Amersham-Pharmacia).

### RT-PCR and miRNA analysis

Total RNA was extracted using the mirVana miRNA™ Isolation Kit (Ambion), treated with RNase-Free DNase (Qiagen) and 1 μg of RNA was used for reverse transcription using Superscript III (Invitrogen) according to the manufacturer’s instructions. Transcript levels were analysed by real-time PCR using SYBR Green Fast PCR master mix (Applied Biosystems) with βeta-actin as an endogenous control.

Mature microRNA levels were measured with TaqMan® MicroRNA Assays (Applied Biosystems) according to the manufacturer’s instructions with RNU6B as the endogenous control.

### Microarray analysis

Human Exon 1.0 ST Affymetrix arrays were used in triplicate. AltAnalyze software was used to calculate SI scores and *P* values [35]. The DAVID Bioinformatics Resources [60, 61] (http://david.abcc.ncifcrf.gov/) were used for GO analysis.

### Trimer analyses

#### Procedure for generating control sequence

To identify significantly differentially expressed exons from the microarray data we used a MiDAS *P* value threshold of <0.05. Hence, for significant unchanged genes, we used a threshold of *P* >0.95. This yielded 17,891 genes with exons not significantly differentially expressed. However, some genes containing exons possibly prone to differential expression may have been weakly expressed and thus undetected in the microarray data. Thus, to find good examples of unchanged exons, we used genes that were moderately expressed in microarray experiments. To further filter our control exons we used data from our RNA-Seq experiments to identify genes that were highly expressed under the same conditions. In our RNA-Seq experiments we had three replicates per condition (control and siSAFB1) for a total of six data sets. We used Tophat [62] to align the reads to the genome and HTSeq [63] to map reads to their corresponding genes. We then set a minimum threshold of 1,000 reads on average per gene per replicate, or 3,000 total reads for each condition. This yielded 2,312 genes with evidence of moderate to high expression in our RNA-Seq data. Of these, 1,498 also had high *P* values in our MiDAS results. We used the SpliceGrapher tool [64] to select our control set of exons, applying the following criteria: to be candidates for exon skipping (cassette exons), exons had to be internal to a transcript, meaning they had to have flanking introns at both the 5’ and 3’ ends. We also enforced two criteria designed to avoid double-counting regions: First, we required that exons be at least 100 nucleotides long to accommodate our regions of interest (the first 50 nt at the 5’ end and the last 50 nt at the 3’ end). Second, we required that the exons be distinct, i.e. non-overlapping. Finally, we required that the exon sequences contain no ambiguous nucleotides (those other than A, C, G or T). From our initial set of 1,498 genes this yielded a pool of 9,478 exon sequences from 1,130 genes for us to use as controls.

### Statistical analysis of key trimers

In our initial analysis, we wished to see whether the trimers of interest (AGA, AAG and GAA) appear more or less frequently in regions surrounding differentially regulated exons than in a set of control exons. Following an approach similar to the RNAmotifs method [31] we marked the positions in each exon where one of our trimers occurs and counted the number of times each position was marked. We then compared these counts to those of our control set and used Fisher’s exact test to identify positions with significant differences (Fig. 5). A key insight provided by the RNAmotifs analysis is the idea that RBPs tend to bind more readily in regions where motifs appear more than once [31]. This may be especially important for short, 3- or 4-nucleotide motifs – individually, they should appear frequently by chance. However, specific pairs may be more rare, particularly in relatively short regions. Hence, we also looked for intron and exon regions where one of our three target motifs appeared a short distance downstream from another of these target motifs within the same region. For example, an exon with an AAG followed by an AGA downstream we would count as a pair motif. To exclude pairs arising from overlapping sequences such as AAGA, we required that downstream motifs start at least k positions downstream of upstream motifs, using k = 3 as the minimum for trimers. Of the regions analyzed, this yielded highly significant over-represented pairs in one particular region: the last 50 nt of downregulated exons. To mitigate the possibility that simple repeats might influence these results, we used RepeatMasker [3] to mask out likely repeat sequences. This yielded five sequences with repeats, two of which had simple (AAG)n repeats, that we removed from our sample. We considered the nine possible distinct trimer combinations plus degenerate combinations of all three trimers. The resulting *P* values ranged from 1.6 × 10^–63^ to 1.8 × 10^–9^ (Fig. 5). The potential importance of this region is underscored by the fact that significant hits in other regions had *P* values well above 1 × 10^–6^.

### Minigene construction and analysis

The NCAM1 minigene was constructed by PCR-amplification of normal human genomic DNA to include exons 7, 8, 9, and 10 with at least 100 bp flanking intronic sequences and cloning into pcDNA3 vector. The minigene comprised exon 7 (143 bp), the 5’ 126 bp and the 3’ 163 bp of intron 7, exon 8 (30 bp), the 5’ 100 bp and the 3’ 142 bp of intron 8, exon 9 (78 bp), all of intron 9 (371 bp), and exon 10 (151 bp). Mutations were introduced to alter all AAG/AGA/GAA trimers in exon 9 by mutating the Gs to Cs. Alternatively, spliced products were detected with the following primers which spanned the following alternative spliced exon junctions: exon 7 to 10: CATCAGCAGCGAAGAAAAGACTC and GGCATGGCTACGCACCAC; exon 8 to 10: GACTCGACCAGAGAAGCAAGAGAC and GCATGGCTACGCACCACC; exon 9 to 10: CAGGCTGGCAGTGCAGGT and GATGCTCTTCAGGGTCAGCG. Hence, the PCR primers were designed to detect only products splicing directly to exon 10.

### Primary hippocampal culture and analysis of dendritic spines

Hippocampal neurons were cultured from E18 Wistar Rat fetuses as described previously [65]. Neurons were transduced with lentivirus expressing EGFP at 3 div (for visualization of spines) and with Adenovirus expressing SAFB1 (or Ad.0 control) at 14 div. Neurons were fixed with 4 % PFA at 18 div. Confocal images were taken with a Leica TCS-SP2-AOBS confocal laser scanning microscope attached to a Leica DM IRE2 inverted epifluorescence microscope. High-resolution images (2048 × 2048 pixels) were taken with a 63× oil-immersion objective as a series of Z stacks at 0.37-μm intervals. All images were averaged four times. Image analysis was performed in ImageJ. Image stacks were projected using the maximum projection algorithm, edges were detected and the image converted to binary. Images were processed to fill and separate individual spines. To assess spine density, the numbers of spines along a given length of dendrite were counted. To measure spine size the cross-sectional area of each spine was measured using the wand tool. For each condition, 50 spines were measured from 24 neurons, taken from three separate cultures. Cumulative plots of spine area were analyzed using the Kolmogorov-Smirnov test.

### Availability of data and materials

The data discussed in this publication have been deposited in NCBI’s Gene Expression Omnibus [66] and are accessible through GEO Series accession number GSE75469.

## Additional files

Additional file 1:
**Crosslinking and immunoprecipitation of SAFB1.** (A) The first panel shows the ^32^P-labeled RNA bound to SAFB1 isolated with or without UV crosslinking separated on a SDS-PAGE gel. (B) Second panel are western blots of extracts from SHSY5Y cells transduced with adenoviral vectors expressing SAFB1 or EGFP. (TIF 4432 kb) Additional file 2:
**Pentamer Z-scores.** (XLSX 75 kb) Additional file 3:
**iCLIP RNA targets with at least five SAFB1 tags.** (XLSX 363 kb) Additional file 4:
**Anti-SAFB1 shRNA mediates the knockdown of SAFB1 and increases MALAT1 expression (** ***P*** **<0.02).** Values are means ± SD of three experiments. Open bars represent scrambled shRNA controls and filled bars represent experiments using the anti-SAFB1 shRNA. (TIF 1666 kb)

## Abbreviations

iCLIP-Seq: Individual-nucleotide resolution cross-linking and immunoprecipitation with deep sequencing
EGFP: Enhanced green fluorescent protein
EJC: Exon junction complex
FDR: False discovery rate
GO: Gene ontology
lncRNA: Long non-coding RNA
ncRNA: Non-coding RNA
RBP: RNA binding protein
SAFB1: Scaffold Attachment Factor B1
SLTM: SAF-like transcription modulator
UTR: Untranslated region

## References

[1] Licatalosi DD, Darnell RB (2010). Applications of next-generation sequencing RNA processing and its regulation: global insights into biological networks. Nat Rev Genet..

[2] Chi SW, Zang JB, Mele A, Darnell RB (2009). Argonaute HITS-CLIP decodes microRNA-mRNA interaction maps. Nature..

[3] Chan CW, Lee YB, Uney J, Flynn A, Tobias JH, Norman M (2007). A novel member of the SAF (scaffold attachment factor)-box protein family inhibits gene expression and induces apoptosis. Biochem J..

[4] Lee YB, Colley S, Norman M, Biamonti G, Uney JB (2007). SAFB re-distribution marks steps of the apoptotic process. Exp Cell Res..

[5] Garee JP, Oesterreich S (2010). SAFB1’s multiple functions in biological control-lots still to be done!. J Cell Biochem..

[6] Baltz AG, Munschauer M, Schwanhausser B, Vasile A, Murakawa Y, Schueler M (2012). The mRNA-bound proteome and its global occupancy profile on protein-coding transcripts. Mol Cell..

[7] Castello A, Fischer B, Eichelbaum K, Horos R, Beckmann BM, Strein C (2012). Insights into RNA biology from an atlas of mammalian mRNA-binding proteins. Cell..

[8] Renz A, Fackelmayer FO (1996). Purification and molecular cloning of the scaffold attachment factor B (SAF-B), a novel human nuclear protein that specifically binds to S/MAR-DNA. Nucleic Acids Res..

[9] Oesterreich S, Lee AV, Sullivan TM, Samuel SK, Davie JR, Fuqua SA (1997). Novel nuclear matrix protein HET binds to and influences activity of the HSP27 promoter in human breast cancer cells. J Cell Biochem..

[10] Weighardt F, Cobianchi F, Cartegni L, Chiodi I, Villa A, Riva S (1999). A novel hnRNP protein (HAP/SAF-B) enters a subset of hnRNP complexes and relocates in nuclear granules in response to heat shock. J Cell Sci..

[11] Kimura H, Sugaya K, Cook PR (2002). The transcription cycle of RNA polymerase II in living cells. J Cell Biol..

[12] Nayler O, Stratling W, Bourquin JP, Stagljar I, Lindemann L, Jasper H (1998). SAF-B protein couples transcription and pre-mRNA splicing to SAR/MAR elements. Nucleic Acids Res..

[13] Townson SM, Dobrzycka KM, Lee AV, Air M, Deng W, Kang K (2003). SAFB2, a new scaffold attachment factor homolog and estrogen receptor corepressor. J Biol Chem..

[14] Arao Y, Kuriyama R, Kayama F, Kato S (2000). A nuclear matrix-associated factor, SAF-B, interacts with specific isoforms of AUF1/hnRNP D. Arch Biochem Biophys..

[15] Denegri M, Chiodi I, Corioni M, Cobianchi F, Riva S, Biamonti G (2001). Stress-induced nuclear bodies are sites of accumulation of pre-mRNA processing factors. Mol Biol Cell..

[16] Townson SM, Dobrzycka KM, Lee AV, Air M, Deng W, Kang K (2003). SAFB2, a new scaffold attachment factor homolog and estrogen receptor corepressor. J Biol Chem.

[17] Li J, Hawkins IC, Harvey CD, Jennings JL, Link AJ, Patton JG (2003). Regulation of alternative splicing by SRrp86 and its interacting proteins. Mol Cell Biol..

[18] Stoss O, Novoyatleva T, Gencheva M, Olbrich M, Benderska N, Stamm S (2004). p59(fyn)-mediated phosphorylation regulates the activity of the tissue-specific splicing factor rSLM-1. Mol Cell Neurosci..

[19] Sergeant KA, Bourgeois CF, Dalgliesh C, Venables JP, Stevenin J, Elliott DJ (2007). Alternative RNA splicing complexes containing the scaffold attachment factor SAFB2. J Cell Sci..

[20] Nikolakaki E, Kohen R, Hartmann AM, Stamm S, Georgatsou E, Giannakouros T (2001). Cloning and characterization of an alternatively spliced form of SR protein kinase 1 that interacts specifically with scaffold attachment factor-B. J Biol Chem..

[21] Hartmann AM, Nayler O, Schwaiger FW, Obermeier A, Stamm S (1999). The interaction and colocalization of Sam68 with the splicing-associated factor YT521-B in nuclear dots is regulated by the Src family kinase p59(fyn). Mol Biol Cell..

[22] Stoss O, Schwaiger FW, Cooper TA, Stamm S (1999). Alternative splicing determines the intracellular localization of the novel nuclear protein Nop30 and its interaction with the splicing factor SRp30c. J Biol Chem..

[23] Stoilov P, Daoud R, Nayler O, Stamm S (2004). Human tra2-beta1 autoregulates its protein concentration by influencing alternative splicing of its pre-mRNA. Hum Mol Genet..

[24] Biamonti G, Vourch C (2010). Nuclear stress bodies. Cold Spring Harb Perspect Biol..

[25] Djebali S, Davis CA, Merkel A, Dobin A, Lassmann T, Mortazavi A (2012). Landscape of transcription in human cells. Nature..

[26] Gerstberger S, Hafner M, Tuschl T (2014). A census of human RNA-binding proteins. Nat Rev Genet..

[27] Castello A, Fischer B, Hentze MW, Preiss T (2013). RNA-binding proteins in Mendelian disease. Trends Genet..

[28] Tollervey JR, Wang Z, Hortobagyi T, Witten JT, Zarnack K, Kayikci M (2011). Analysis of alternative splicing associated with aging and neurodegeneration in the human brain. Genome Res..

[29] Ule J, Jensen KB, Ruggiu M, Mele A, Ule A, Darnell RB (2003). CLIP identifies Nova-regulated RNA networks in the brain. Science..

[30] Konig J, Zarnack K, Rot G, Curk T, Kayikci M, Zupan B (2010). iCLIP reveals the function of hnRNP particles in splicing at individual nucleotide resolution. Nat Struct Mol Biol..

[31] Cereda M, Pozzoli U, Rot G, Juvan P, Schweitzer A, Clark T (2014). RNAmotifs: prediction of multivalent RNA motifs that control alternative splicing. Genome Biol..

[32] Gnanapavan S, Giovannoni G (2013). Neural cell adhesion molecules in brain plasticity and disease. Mult Scler Relat Disord..

[33] Zhang W, Xiao MS, Ji S, Tang J, Xu L, Li X (2014). Promoter variant rs2301228 on the neural cell adhesion molecule 1 gene confers risk of schizophrenia in Han Chinese. Schizophr Res..

[34] Vawter MP, Frye MA, Hemperly JJ, VanderPutten DM, Usen N, Doherty P (2000). Elevated concentration of N-CAM VASE isoforms in schizophrenia. J Psychiatr Res..

[35] Emig D, Salomonis N, Baumbach J, Lengauer T, Conklin BR, Albrecht M (2010). AltAnalyze and DomainGraph: analyzing and visualizing exon expression data. Nucleic Acids Res..

[36] Atz ME, Rollins B, Vawter MP (2007). NCAM1 association study of bipolar disorder and schizophrenia: polymorphisms and alternatively spliced isoforms lead to similarities and differences. Psychiatr Genet..

[37] Wang KS, Liu XF, Aragam N (2010). A genome-wide meta-analysis identifies novel loci associated with schizophrenia and bipolar disorder. Schizophr Res..

[38] Millar JK, Pickard BS, Mackie S, James R, Christie S, Buchanan SR (2005). DISC1 and PDE4B are interacting genetic factors in schizophrenia that regulate cAMP signaling. Science..

[39] Tollervey JR, Curk T, Rogelj B, Briese M, Cereda M, Kayikci M (2011). Characterizing the RNA targets and position-dependent splicing regulation by TDP-43. Nat Neurosci..

[40] Anko ML, Muller-McNicoll M, Brandl H, Curk T, Gorup C, Henry I (2012). The RNA-binding landscapes of two SR proteins reveal unique functions and binding to diverse RNA classes. Genome Biol..

[41] Wang X, Juan L, Lv J, Wang K, Sanford JR, Liu Y (2011). Predicting sequence and structural specificities of RNA binding regions recognized by splicing factor SRSF1. BMC Genomics.

[42] Jovicic A, Roshan R, Moisoi N, Pradervand S, Moser R, Pillai B (2013). Comprehensive expression analyses of neural cell-type-specific miRNAs identify new determinants of the specification and maintenance of neuronal phenotypes. J Neurosci..

[43] He L, Thomson JM, Hemann MT, Hernando-Monge E, Mu D, Goodson S (2005). A microRNA polycistron as a potential human oncogene. Nature..

[44] Mogilyansky E, Rigoutsos I (2013). The miR-17/92 cluster: a comprehensive update on its genomics, genetics, functions and increasingly important and numerous roles in health and disease. Cell Death Differ..

[45] Sauliere J, Murigneux V, Wang Z, Marquenet E, Barbosa I, Le Tonqueze O (2012). CLIP-seq of eIF4AIII reveals transcriptome-wide mapping of the human exon junction complex. Nat Struct Mol Biol..

[46] Long JC, Caceres JF (2009). The SR protein family of splicing factors: master regulators of gene expression. Biochem J..

[47] Leussis MP, Madison JM, Petryshen TL (2012). Ankyrin 3: genetic association with bipolar disorder and relevance to disease pathophysiology. Biol Mood Anxiety Disord..

[48] McClay JL, Adkins DE, Aberg K, Stroup S, Perkins DO, Vladimirov VI (2011). Genome-wide pharmacogenomic analysis of response to treatment with antipsychotics. Mol Psychiatry..

[49] Sato J, Shimazu D, Yamamoto N, Nishikawa T (2008). An association analysis of synapse-associated protein 97 (SAP97) gene in schizophrenia. J Neural Transm..

[50] Ruediger S, Spirig D, Donato F, Caroni P (2012). Goal-oriented searching mediated by ventral hippocampus early in trial-and-error learning. Nat Neurosci..

[51] Hoepfner S, Severin F, Cabezas A, Habermann B, Runge A, Gillooly D (2005). Modulation of receptor recycling and degradation by the endosomal kinesin KIF16B. Cell..

[52] Loo SK, Shtir C, Doyle AE, Mick E, McGough JJ, McCracken J (2012). Genome-wide association study of intelligence: additive effects of novel brain expressed genes. J Am Acad Child Adolesc Psychiatry.

[53] Patodia S, Raivich G (2012). Role of transcription factors in peripheral nerve regeneration. Front Mol Neurosci..

[54] Arinami T, Ohtsuki T, Ishiguro H, Ujike H, Tanaka Y, Morita Y (2005). Genomewide high-density SNP linkage analysis of 236 Japanese families supports the existence of schizophrenia susceptibility loci on chromosomes 1p, 14q, and 20p. Am J Hum Genet..

[55] Bassett AS, Costain G, Fung WL, Russell KJ, Pierce L, Kapadia R (2010). Clinically detectable copy number variations in a Canadian catchment population of schizophrenia. J Psychiatr Res..

[56] Hamshere ML, Bennett P, Williams N, Segurado R, Cardno A, Norton N (2005). Genomewide linkage scan in schizoaffective disorder: significant evidence for linkage at 1q42 close to DISC1, and suggestive evidence at 22q11 and 19p13. Arch Gen Psychiatry..

[57] Konig J, Zarnack K, Rot G, Curk T, Kayikci M, Zupan B, et al. iCLIP--transcriptome-wide mapping of protein-RNA interactions with individual nucleotide resolution. J Vis Exp. 2011;(50). doi:10.3791/2638. 10.3791/2638 PMC3169244 21559008

[58] Broughton JP, Pasquinelli AE (2013). Identifying Argonaute binding sites in Caenorhabditis elegans using iCLIP. Methods..

[59] Wang Z, Kayikci M, Briese M, Zarnack K, Luscombe NM, Rot G (2010). iCLIP predicts the dual splicing effects of TIA-RNA interactions. PLoS Biol..

[60] da Huang W, Sherman BT, Lempicki RA (2009). Systematic and integrative analysis of large gene lists using DAVID bioinformatics resources. Nat Protoc..

[61] da Huang W, Sherman BT, Tan Q, Kir J, Liu D, Bryant D (2007). DAVID Bioinformatics Resources: expanded annotation database and novel algorithms to better extract biology from large gene lists. Nucleic Acids Res..

[62] Trapnell C, Pachter L, Salzberg SL (2009). TopHat: discovering splice junctions with RNA-Seq. Bioinformatics..

[63] Anders S, Pyl PT, Huber W (2014). HTSeq-a Python framework to work with high-throughput sequencing data. Bioinformatics..

[64] Rogers MF, Thomas J, Reddy AS, Ben-Hur A (2012). SpliceGrapher: detecting patterns of alternative splicing from RNA-Seq data in the context of gene models and EST data. Genome Biol..

[65] Howarth JL, Glover CP, Uney JB (2009). HSP70 interacting protein prevents the accumulation of inclusions in polyglutamine disease. J Neurochem..

[66] Edgar R, Domrachev M, Lash AE (2002). Gene Expression Omnibus: NCBI gene expression and hybridization array data repository. Nucleic Acids Res..

