# Supplementary figures and images for: iCLIP identifies novel roles for SAFB1 in regulating RNA processing and neuronal function
Source: BMC Biol. 2015 Dec 22;13:111. doi: 10.1186/s12915-015-0220-7 (PMC4689037; doi:10.1186/s12915-015-0220-7)

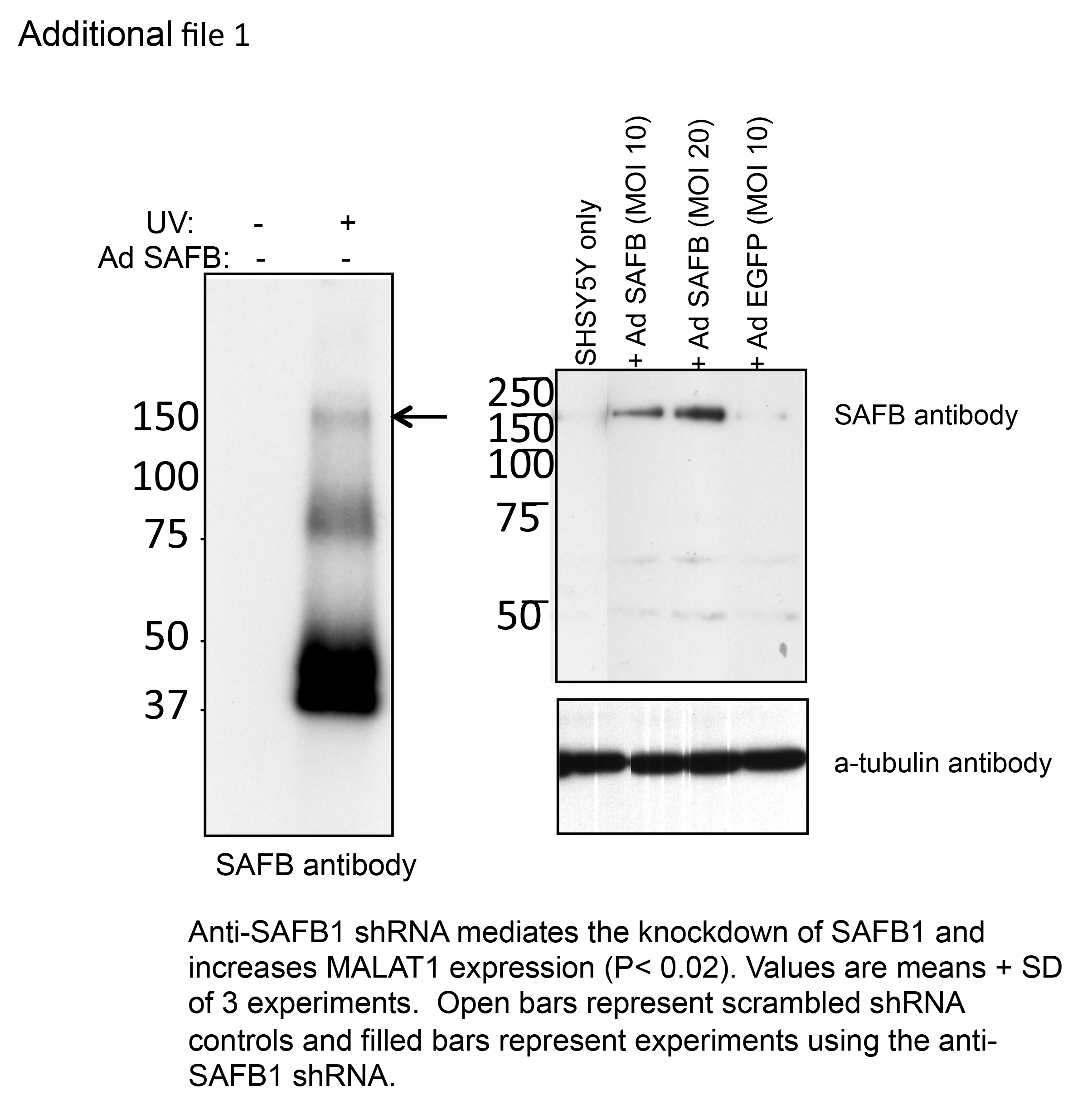

Supplement: Additional file 1: — Crosslinking and immunoprecipitation of SAFB1. (A) The first panel shows the 32P-labeled RNA bound to SAFB1 isolated with or without UV crosslinking separated on a SDS-PAGE gel. (B) Second panel are western blots of extracts from SHSY5Y cells transduced with adenoviral vectors expressing SAFB1 or EGFP. (TIF 4432 kb) [file 12915_2015_220_MOESM1_ESM.tif]

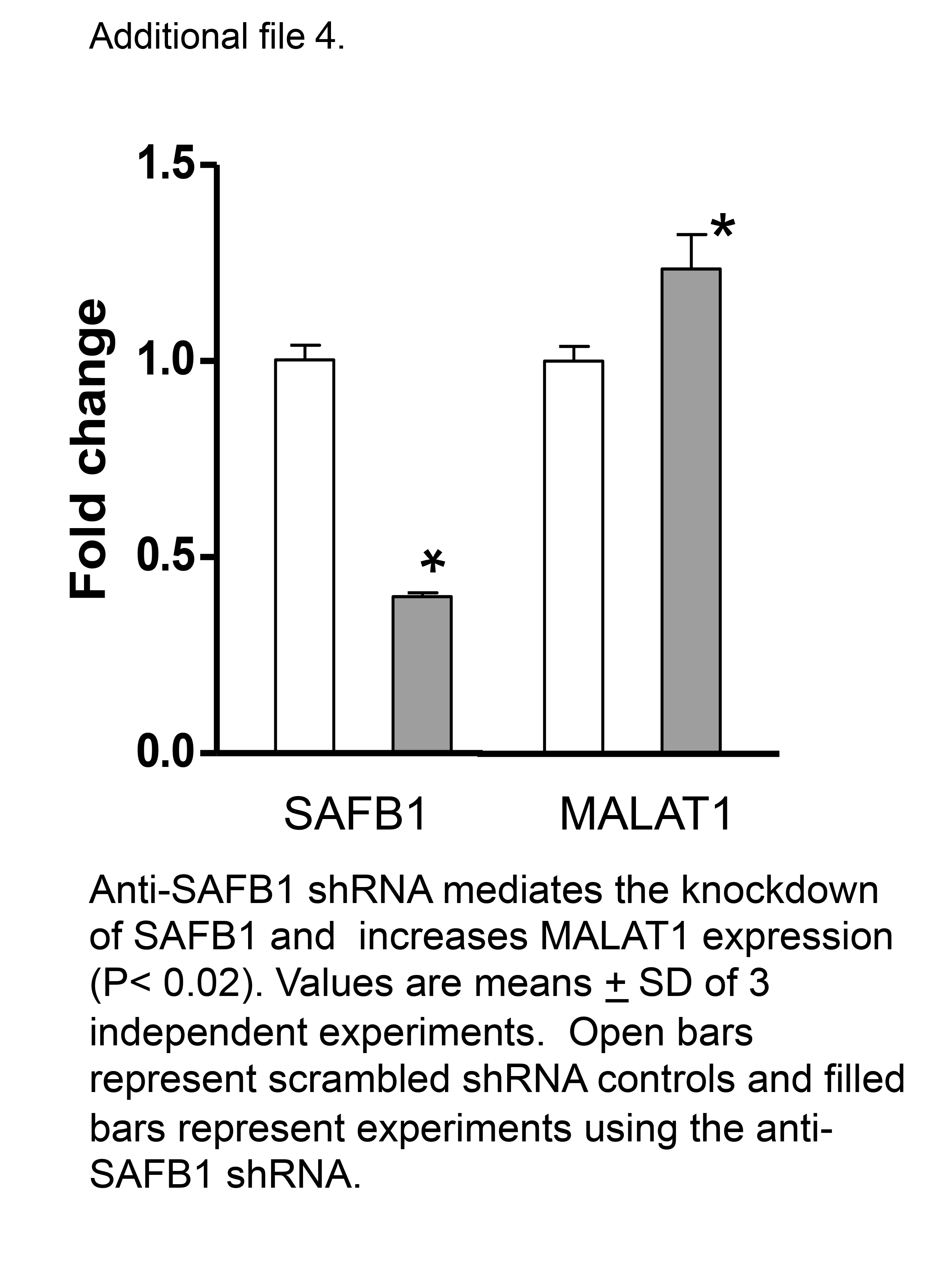

Supplement: Additional file 4: — Anti-SAFB1 shRNA mediates the knockdown of SAFB1 and increases MALAT1 expression ( P <0.02). Values are means ± SD of three experiments. Open bars represent scrambled shRNA controls and filled bars represent experiments using the anti-SAFB1 shRNA. (TIF 1666 kb) [file 12915_2015_220_MOESM4_ESM.tif]
